# Longitudinal evaluation of serum periostin levels in patients after large-artery atherosclerotic stroke: A prospective observational study

**DOI:** 10.1038/s41598-018-30121-5

**Published:** 2018-08-06

**Authors:** Xinwei He, Yuyan Bao, Yuguang Shen, En Wang, Weijun Hong, Shaofa Ke, Xiaoping Jin

**Affiliations:** Department of Neurology, Taizhou Hospital, Wenzhou Medical University, Zhejiang, 317000 China

## Abstract

Increasing evidence supports the involvement of periostin in the pathophysiological processes of stroke and atherosclerosis. The aim of this study was to assess circulating periostin levels at different times after large-artery atherosclerotic (LAA) stroke and their association with stroke. Serum periostin levels were measured using enzyme-linked immunosorbent assay on day 1 in 162 patients with LAA stroke and in 108 age- and sex-matched controls, on day 6 after stroke in 134 patients, and during the 4th week after stroke in 46 of the 162 patients. Stroke severity was determined using the National Institutes of Health Stroke Scale (NIHSS), and the stroke volume was measured. Outcome at 3 months was measured using the modified Rankin Scale (mRS). Our results indicated that periostin levels increased significantly on day 6 after stroke, and this increasing trend persisted for at least 4 weeks after the event. In addition, the increase in periostin levels was positively correlated with the NIHSS scores and stroke volume, but not with the mRS scores after adjusting for the NIHSS scores. In conclusion, these findings suggest that the increase in serum periostin levels observed after stroke may be associated with the stroke severity in patients with LAA stroke.

## Introduction

Stroke has become the leading cause of mortality and adult disability in China^1^. Ischaemic stroke (IS) accounts for approximately 80% of all stroke cases and causes a tremendous burden on health resources and families^2^. IS is characterized by the disruption of cerebral blood flow, leading to dysfunction of the affected area^2^. Early evaluations of severity and predictions of outcome are considered essential and critical in the treatment and care of patients with IS.

Periostin is a secreted extracellular matrix protein with known functions in osteology, oncology, and the cardiovascular and respiratory systems^3,4^. In general, periostin is expressed at low levels in tissues; however, periostin levels can increase rapidly under various adverse conditions^3,5^. Periostin expression is upregulated in the early stage of fracture healing and plays an important role in periosteal callus formation^6^. Additionally, periostin levels increase during the tissue remodelling process involved in wound repair, and periostin knock-out delays cutaneous wound healing^7,8^. Periostin has been shown to be rapidly secreted into infarcted tissue after acute myocardial infarction and is essential for the repair process^9,10^. Furthermore, previous studies have revealed the neuroprotective and neurogenic effects of periostin on IS models and cultured neurons^11,12^.

Recently, the capacity to measure serum periostin levels has prompted notable efforts to evaluate the severity and prognosis of specific diseases^13–15^. However, data on the periostin levels in patients with acute IS are not available.

In the present study, we compared the serum periostin levels measured in patients with large-artery atherosclerotic (LAA) stroke on day 1 and day 6 and during the 4th week after IS and the levels measured in age- and sex-matched controls. We also investigated the associations of periostin levels and changes in periostin levels with stroke severity and short-term outcomes after IS.

## Results

### Study population

**Figure 1:**
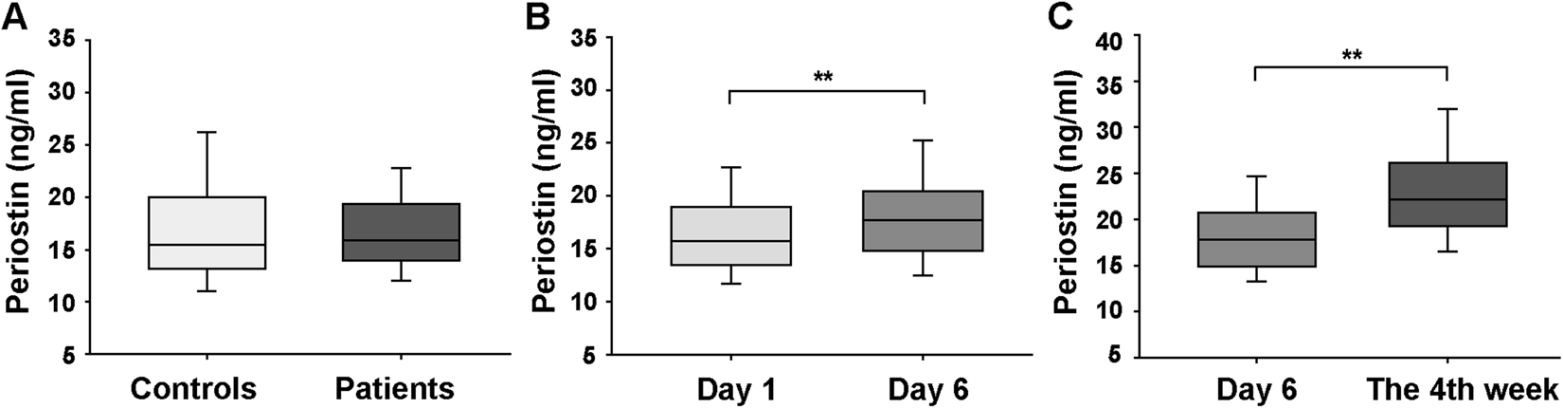
Serum periostin levels in controls and patients at different time points. (**A**) Comparison of the periostin levels in 108 controls and 162 patients. (**B**) Comparison of periostin levels on days 1 and 6 after ischaemic stroke (134 patients). (**C**) Comparison of periostin levels on day 6 and the 4th week after stroke (46 patients). Student’s *t*-test (**A**) or the Wilcoxon signed-rank test (**B**,**C**) was used to assess differences. ^**^p < 0.01. Boxes represent IQRs, with horizontal lines representing the medians, the lower and upper whiskers representing the 25th and 75th percentiles, and the error bars extending below and above the boxes representing the 10th and 90th percentiles, respectively.

The basic characteristics of the study subjects are shown in Table 1. Patients with LAA stroke had higher systolic blood pressure (SBP), fasting blood glucose (FBG) level, homocysteine level, and fibrinogen level and made up a larger percentage of smokers but had a lower percentage of dyslipidaemia than control subjects. No differences in the serum periostin levels were observed between the patients and controls (17.05 ± 4.95 ng/ml vs. 17.46 ± 6.60 ng/ml, *p* = 0.583, Fig. 1A).

**Table 1:** Baseline characteristics of the study population.

Characteristic	Patients (n = 162)	Controls (n = 108)	*p* value
Age (years)	71.7 ± 8.8	71.4 ± 9.5	0.816
Male (%)	105 (64.8)	70 (64.8)	1.000
SBP (mmHg)	152.9 ± 23.7	138.7 ± 19.3	<0.001
DBP (mmHg)	77.7 ± 12.8	78.8 ± 8.5	0.407
BMI (kg/m^2^)	23.1 ± 3.3	23.5 ± 3.6	0.371
FBG (mmol/L)	5.30 (4.76, 6.07)	5.05 (4.56, 5.74)	0.016
TG (mmol/L)	1.34 (1.00, 1.71)	1.41 (1.06, 1.97)	0.082
TC (mmol/L)	4.42 (3.84, 5.17)	4.35 (3.63, 5.25)	0.401
HDL-C (mmol/L)	1.18 ± 0.22	1.18 ± 0.23	0.946
LDL-C (mmol/L)	2.54 (2.13, 3.26)	2.69 (1.99, 3.36)	0.506
HbA_1C_ (%)	5.9 (5.5, 6.3)	5.7 (5.4, 6.2)	0.079
Homocysteine (μmol/L)	13.0 (11.0, 17.2)	11.8 (9.4, 14.8)	0.006
Serum creatinine (μmol/L)	70.5 ± 17.2	70.0 ± 16.2	0.829
Fibrinogen (g/L)	3.62 (3.19, 4.42)	3.09 (2.79, 3.34)	<0.001
Hs-CRP (mg/L)	3.4 (2.1, 5.9)	2.1 (1.7, 3.3)	<0.001
Hypertension	119 (73.5)	75 (69.4)	0.473
Diabetes mellitus	42 (25.9)	22 (20.4)	0.293
Dyslipidaemia	67 (41.4)	62 (57.4)	0.010
Smokers	73 (45.1)	22 (20.4)	<0.001
Alcohol consumers	32 (19.8)	17 (15.7)	0.402
Hypertension med use	40 (33.6^a^)	31 (41.3^a^)	0.277
Diabetes med use	21 (50.0^b^)	14 (63.6^b^)	0.615
NIHSS on day 1	4 (2, 8)	/	/
mRS scores	3 (2, 3)	/	/

Continuous variables are expressed as the means ± standard deviations (SDs) or the medians (interquartile ranges). Categorical values are given as frequencies (percentages).

^a^Represents the percentage in the hypertension population.

^b^Represents the percentage in the diabetic population.

Abbreviations: LAA stroke, large-artery atherosclerotic stroke; SBP, systolic blood pressure; DBP, diastolic blood pressure; BMI, body mass index; FBG, fasting blood glucose; TG, triglycerides; TC, total cholesterol; HDL-C, high-density lipoprotein cholesterol; LDL-C, low-density lipoprotein cholesterol; HbA_1C_, glycated haemoglobin; hs-CRP, high-sensitivity C-reactive protein; NIHSS, National Institutes of Health Stroke Scale; mRS, modified Rankin Scale.

### Serum periostin levels continuously increased after stroke for at least 4 weeks

The analysis of the time course of serum periostin levels in 134 patients showed higher periostin levels on day 6 after IS than on day 1 (day 6: 17.74 (14.81, 20.38) ng/ml vs. day 1: 15.73 (13.45, 18.94) ng/ml, *p* < 0.001, Fig. 1B). Furthermore, we analysed the periostin levels in 46 patients during the 4th week after IS and found that the periostin levels continuously increased at least up to the 4th week after the event (the 4th week: 22.11 (19.26, 26.11) ng/ml vs. day 6: 17.79 (14.90, 20.71) ng/ml, *p* < 0.001, Fig. 1C).

### The increase in periostin levels observed after stroke was associated with stroke severity

**Figure 2:**
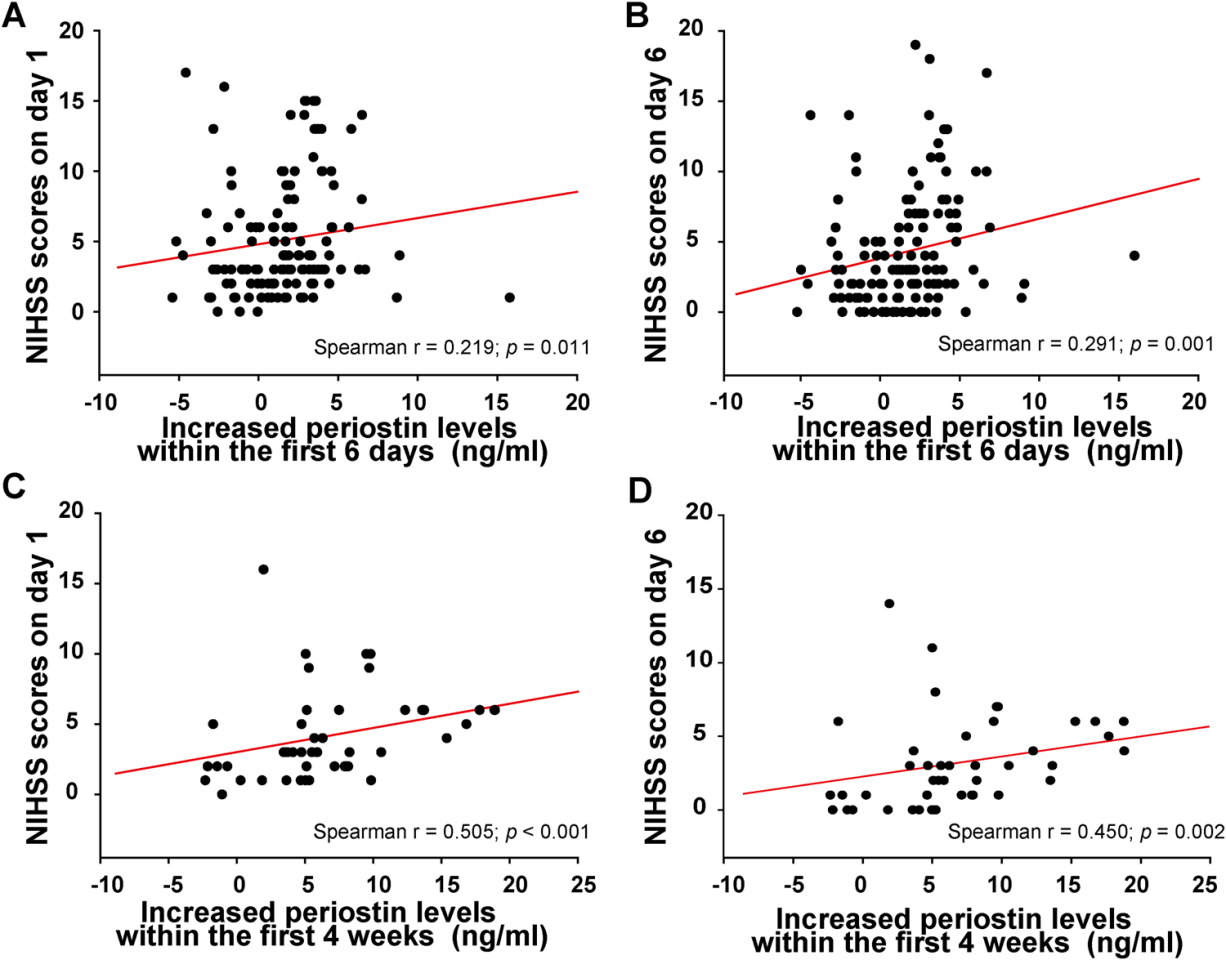
Correlation between the increased periostin levels and National Institutes of Health Stroke Scale (NIHSS) scores. (**A**,**B**) Correlation between the increased periostin levels within the first 6 days and the NIHSS scores on day 1 and day 6. (**C**,**D**) Correlation between the increased periostin levels within the first 4 weeks and the NIHSS scores on day 1 and day 6. Abbreviations: NIHSS, National Institutes of Health Stroke Scale.

The increase in periostin levels observed during the first 6 days was correlated with National Institutes of Health Stroke Scale (NIHSS) scores on both day 1 (*r* = 0.219, *p* = 0.011, Fig. 2A) and day 6 (*r* = 0.291, *p* = 0.001, Fig. 2B), but not with stroke volume (*r* = −0.026, *p* = 0.771). Furthermore, the increase in periostin levels observed from day 1 to the 4th week was correlated with the NIHSS scores on day 1 (*r* = 0.505; *p* < 0.001, Fig. 2C) and day 6 (*r* = 0.450, *p* = 0.002, Fig. 2D), along with the stroke volume (*r* = 0.352, *p* = 0.022).

**Figure 3:**
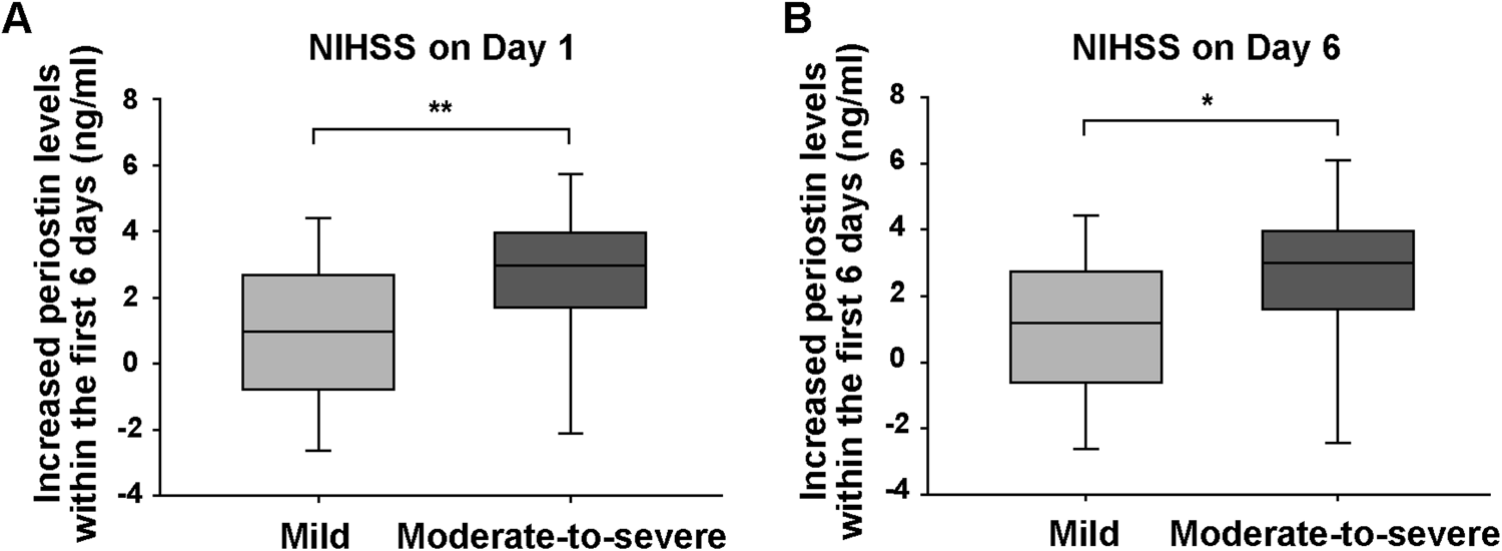
The increased periostin levels within the first 6 days were grouped according to the NIHSS scores. (**A**) NIHSS scores on day 1 after stroke. (**B**) NIHSS scores on day 6 after stroke. Mild stroke, NIHSS score < 8; moderate-to-severe stroke, NIHSS score ≥8. Differences were compared using the Mann-Whitney *U* test. ^*^p < 0.05, ^**^p < 0.01. Boxes represent IQRs, with horizontal lines representing the medians, the lower and upper whiskers representing the 25th and 75th percentiles, and the error bars extending below and above the boxes represent the 10th and 90th percentiles, respectively.

Additionally, the increase in periostin levels observed during the first 6 days was higher in patients with a moderate-to-severe stroke than in patients with a mild stroke (NIHSS on day 1, *p* = 0.004, Fig. 3A; NIHSS on day 6, *p* = 0.015, Fig. 3B).

### No associations were observed between serum periostin levels and functional outcomes after stroke

No differences in periostin levels (either on day 1 or day 6) were observed between patients with good outcomes and patients with poor outcomes (16.70 ± 5.04 ng/ml vs. 17.36 ± 4.53 ng/ml, *p* = 0.403). In addition, the change in periostin levels was positively correlated with the modified Rankin Scale (mRS) scores; however, after further adjustment for the NIHSS score, this relationship disappeared.

## Discussion

In this study, we investigated the post-stroke time course of serum periostin levels. The serum periostin levels observed on day 1 after LAA stroke did not change compared with those of the healthy controls but did increase significantly on day 6 after the event. This increasing trend persisted for at least 4 weeks after stroke. The increased periostin levels were positively correlated with the NIHSS scores and stroke volume but were not correlated with the mRS scores after adjusting for the NIHSS scores.

To the best of our knowledge, the present study constitutes the first evaluation of the serum periostin levels in humans after IS. Previous studies have demonstrated that the periostin levels in the brain increase during the acute, subacute, and chronic stages of IS in animal models^11,12^. Periostin levels increase 24 h after cerebral ischaemia and remain high for up to 28 days afterward. Based on the results from the present study, serum periostin levels were stable on day 1 and then continued to increase through the 4th week after IS, similar to the changes in the intracranial levels observed in the animal models described above.

In addition, a recent study reported that serum periostin levels were significantly increased in patients with intracerebral haemorrhage (ICH) within 6 h after ICH compared with controls, and its levels were consistently increased within almost 24 h after ICH^16^. It seems that the uptrend of periostin levels is consistent after onset between ischaemic and haemorrhagic stroke, although the rising speed is different.

Previous studies found that increased periostin levels were associated with clinical severity and poor outcome in patients with traumatic brain injury^17^, aneurysmal subarachnoid haemorrhage^18^, and ICH^16^. In the present study, the increased periostin levels observed in the first 6 days or within 4 weeks after IS were correlated with the NIHSS scores, indicating that the new accumulation of periostin in the circulation might reflect the degree of IS severity.

According to the results from animal experiments, periostin levels initially increase in the peri-ischaemic and ischaemic regions after IS; however, between days 14 and 28, periostin is strongly expressed in the peri-infarct region, rather than the infarct core^11,12^. The specific temporal and spatial patterns of periostin expression observed after IS might account for the relationship between the changes in periostin levels and stroke volume observed within 4 weeks after IS, but not in the first 6 days after IS.

Accumulating evidence has indicated that periostin is a neurite outgrowth-promoting factor. Periostin promotes the proliferation and differentiation of neural stem cells in the brain after ischaemic or haemorrhagic injury^19^, which may rescue neuronal loss and restore brain function^20^. An injection of periostin into the lateral ventricles of hypoxic-ischaemic rats was recently shown to significantly improve spatial learning and memory, indicating that periostin may reverse cognitive deficits after IS^19^. Thus, circulating periostin levels may be associated with post-stroke cognitive impairment rather than the disability.

Angiogenesis plays an important role in the formation of new brain microvessels after IS^21^. Periostin has been consistently shown to promote neovascularization and angiogenesis^9,10,22–25^. Thus, in addition to the neuroprotective effects of periostin, the increase in serum periostin levels is likely involved in the vasculogenesis and angiogenesis of microvessels that occurs to protect against ischaemic injury after IS.

Regarding inflammation, periostin has been recognized as an inflammatory effector in asthma^26^. A previous study found that periostin was upregulated in the cerebral cortex after experimental subarachnoid haemorrhage in mice and was responsible for early blood-brain barrier disruption and brain oedema formation through activating downstream signalling pathways^27^, possibly because different periostin splicing variants show different functions depending on specific diseases.

Periostin continued to be expressed up to 28 days after cerebral ischaemia in various cells, such as astrocytes/microglia and neuronal progenitor cells^11^. There is a possibility that periostin generated in the brain may leak into the circulation due to the breakdown of the blood-brain barrier that occurs after IS. However, the serum periostin levels were persistently increased for at least 4 weeks, whereas the damage to the blood-brain barrier gradually recovered. Cerebral ischaemia triggers biological reactions in both the affected brain region and the circulation. Therefore, it is unclear whether the increased periostin levels were predominantly derived from the breakdown of the blood-brain barrier or from other specific tissues or were due to overlap from both sources. Indeed, the sources of circulating periostin may include mesenchymal cells, such as bone, myocardium, and skin cells^28–30^.

Furthermore, studies have reported that the intracerebroventricular administration of recombinant periostin exerts a protective effect after IS^12,19^. Based on the consistent changes in periostin levels in the circulation and brain after IS, recombinant periostin may have therapeutic effects when administered via the circulation. Indeed, the application of recombinant periostin via the circulation improves cutaneous wounds, limb ischaemia, and myocardial infarction^9,10,25^.

The daily routines and lifestyles of patients tend to change after IS, at least during the hospitalization and subsequent rehabilitation periods. Notably, periostin is required for the integrity and function of periodontium and bones, and the expression of periostin in these tissues increases in response to mechanical loading (such as occlusal loading, axial compression, and exercise) and decreases with reduced loading^31,32^. During the acute phase and convalescence after IS, the motor function and exercise intensity of patients are relatively weak, which may attenuate the expression of periostin in bones; however, whether mechanical loading can affect periostin in the circulation has not been elucidated.

The present study had several limitations. First, our study demonstrated a direct correlation of the increase in periostin levels with NIHSS scores and stroke volume, while the correlation coefficients reflected weak or moderate correlation. Thus, the explanation needs to be more rigorous, and further multi-centre studies with larger sample sizes are needed to confirm our findings. Second, this work lacked long-term clinical prognostic data and post-stroke cognition evaluations, which may be associated with serum periostin levels or the changes in periostin levels. Third, although various alternatively spliced isoforms of periostin have been described in humans, little is known about their specific and even opposing functions^11,12^. Indeed, the enzyme-linked immunosorbent assay (ELISA) kit used in our study detects all periostin isoforms present in the circulation. Hence, further studies are needed to develop new ELISA kits that distinguish the splicing variants. Finally, considering the clinical relevance of our study, we did not completely elucidate the pathophysiological mechanism. Nevertheless, this study extended the knowledge of the role of periostin in patients after IS.

In conclusion, we provided preliminary evidence that serum periostin levels continuously increase for at least 4 weeks after LAA stroke. The increased periostin levels are associated with stroke severity. However, further studies are needed to determine whether and how circulating periostin is involved in pathophysiological processes after LAA stroke. Further research should focus on clarifying the effects and sources of the increased circulating periostin levels and may also help us develop a novel clinical therapy for IS.

## Methods

### Study subjects

The study included 162 LAA stroke patients, who were consecutively recruited from the Department of Neurology at Taizhou Hospital, Zhejiang, China, from June 2014 to May 2015. Acute ischaemic stroke was diagnosed according to World Health Organization criteria^33^. LAA stroke was defined according to the Trial of Org 10172 in Acute Stroke Treatment (TOAST) criteria^34^, reviewed by two neurological physicians.

The following inclusion criteria were applied: first-time stroke, admission within 24 h after the event, and without thrombolysis or interventional therapy. The following exclusion criteria were applied: any previous history of stroke (including ischaemic stroke, intracerebral haemorrhage, or subarachnoid haemorrhage); severe coronary heart disease or arrhythmias; ischaemic peripheral arterial disease; chronic inflammatory, autoimmune, or haematological diseases; severe hepatic or renal insufficiencies or thyroid diseases; history of cancers; and previous surgical history (3 months).

As a control group, 108 age-matched (blocks of 5 years) and sex-matched healthy individuals were recruited. They were free of the exclusion criteria.

Study was approved by the Medical Ethics Committee of Taizhou Hospital. All procedures were performed in accordance with the ethical guidelines of the 1975 Declaration of Helsinki. Written informed consent was provided by all participants before enrolment in the present study.

### Clinical protocol

Stroke severity was assessed using the infarct volume and the NIHSS^35^. Mild stroke was defined as an NIHSS score < 8, and moderate-to-severe stroke was defined as an NIHSS score ≥ 8, consistent with the existing literature^36,37^. The infarct volume was evaluated in 151 patients using the initial diffusion-weighted imaging lesion volume and was calculated as the sum of the infarcted area of every slice multiplied by the slice thickness, based on several previous studies^38,39^. Structured telephone follow-ups were conducted by one doctor who was blinded to the patients’ clinical information. A poor outcome was defined as a mRS score >2 at 3 months after stroke, as usual^40–42^.

**Figure 4:**
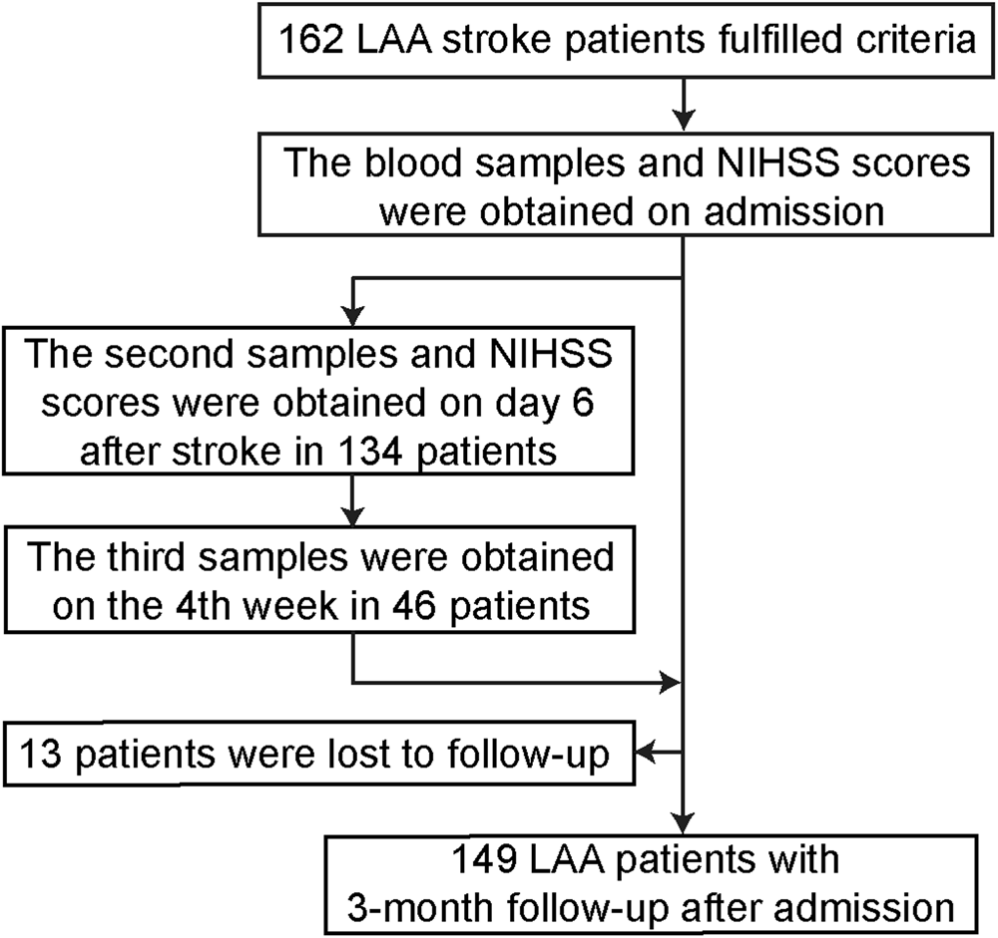
Study flow chart. Abbreviations: LAA stroke, large-artery atherosclerotic stroke; NIHSS, National Institutes of Health Stroke Scale; mRS, modified Rankin Scale.

A flow chart of the study is shown in Fig. 4. Blood samples were collected on the morning after an overnight fast. The second blood samples were collected from 134 patients on day 6 after stroke. In the 162 LAA stroke patients, 5 patients died and 23 patients had been discharged or transferred within the first 6 days following admission. The characteristics of these subjects were not significantly different from those of the patients as a group (Supplementary Table S1). Furthermore, the third blood samples of 46 patients (from the 134 patients) were obtained on the 4th week during their reviews. The characteristics of these subjects were similar to those of the 162 patients, except for higher fibrinogen (Supplementary Table S2). The control subjects had a single blood sample on admission.

### Baseline clinical data collection and definition

Baseline data, including demographic data, conventional risk factors, health history, and medications, were obtained from all participants. Height and weight were measured to calculate the body mass index.

Assessment of baseline vascular risk factors included hypertension (SBP/DBP ≥ 140/90 mm Hg based on the average of two measurements on admission, history of hypertension, or the use of antihypertensive medications), diabetes mellitus (FBG ≥ 7 mmol/L, diabetes history, or the use of antidiabetic treatments), dyslipidaemia (serum triglyceride ≥1.69 mmol/L, low-density lipoprotein ≥3.37 mmol/L, high-density lipoprotein cholesterol ≤0.91 mmol/L, or the use of lipid-lowering drugs), smokers (those who were currently smoking or had quit within the past year), and alcohol consumers (those who had moderate or heavy alcohol consumption (≥2 standard alcoholic beverages per day), as referenced in previous literature^43^.

### Sample quantification and laboratory testing

Samples were stored at −80 °C before testing. Periostin levels were measured using a commercially available ELISA kit (BioVendor, Brno, Czech Republic; catalogue number RAG019R). The inter- and intra-assay coefficients of variation were 4.54–9.90% and 6.22–8.60%, respectively. The mean minimum detectable dose was 15 pg/ml.

Biochemical measurements (e.g., FBG) were performed at the clinical laboratory in our hospital.

### Statistical analysis

The Kolmogorov-Smirnov test was used for normality testing. Continuous data are expressed as the means ± standard deviations (SDs) or medians (interquartile ranges, IQRs). Categorical data are presented as counts and percentages. Comparisons between groups were made using Student’s *t*-test, the Mann-Whitney *U* test, or Pearson’s chi-squared statistic, as appropriate. Differences in the periostin levels at different time points were assessed using the Wilcoxon signed-rank test. Pearson’s correlation test or Spearman’s rank correlation test was performed as appropriate. Data were analysed using SPSS version 18.0 (SPSS Inc., Chicago, IL, USA). *p* < 0.05 was considered statistically significant.

### Data availability

All data are fully available from the corresponding author on reasonable request.

## Electronic supplementary material

Supplementary Information
