# Supplementary material for: Longitudinal evaluation of serum periostin levels in patients after large-artery atherosclerotic stroke: A prospective observational study
Source: Sci Rep. 2018 Aug 6;8:11729. doi: 10.1038/s41598-018-30121-5 (PMC6079094; doi:10.1038/s41598-018-30121-5)
Supplement: Supplementary file 1 — Supplementary Information [file 41598_2018_30121_MOESM1_ESM.docx]

**Supplementary Information**

**Title**

Longitudinal evaluation of serum periostin levels in patients after large-artery atherosclerotic stroke: A prospective observational study

**Author names and affiliations**

Xinwei He, Yuyan Bao, Yuguang Shen, En Wang, Weijun Hong, Shaofa Ke, Xiaoping Jin *

Department of Neurology, Taizhou Hospital, Wenzhou Medical University, Taizhou, 317000, China

*corresponding author, E-mail: [hhh841062205@aliyun.com](mailto:hhh841062205@aliyun.com)

| Characteristic | Patients analysed periostin levels twice  (n = 134) | All stroke patients  (n = 162) | *p* value |
| --- | --- | --- | --- |
| Age (years) | 71.4 ± 8.3 | 71.7 ± 8.8 | 0.763 |
| Male (%) | 88 (65.7) | 105 (64.8) | 0.878 |
| SBP (mmHg) | 152.8 ± 24.1 | 152.9 ± 23.7 | 0.975 |
| DBP (mmHg) | 77.9 ± 12.6 | 77.7 ± 12.8 | 0.889 |
| BMI (kg/m^2^) | 23.2 ± 3.4 | 23.1 ± 3.3 | 0.844 |
| FBG (mmol/L) | 5.31 (4.75, 7.29) | 5.30 (4.76, 6.07) | 0.625 |
| TG (mmol/L) | 1.31 (1.00, 1.72) | 1.34 (1.00, 1.71) | 0.831 |
| TC (mmol/L) | 4.34 (3.79, 5.15) | 4.42 (3.84, 5.17) | 0.474 |
| HDL-C (mmol/L) | 1.16 ± 0.22 | 1.18 ± 0.22 | 0.469 |
| LDL-C (mmol/L) | 2.50 (2.11, 3.26) | 2.54 (2.13, 3.25) | 0.643 |
| HbA_1C_ (%) | 5.9 (5.5, 6.4) | 5.9 (5.5, 6.3) | 0.884 |
| Homocysteine (μmol/L) | 12.9 (11.0, 16.4) | 13.0 (11.0, 17.2) | 0.976 |
| Serum creatinine (μmol/L) | 69.8 ± 16.1 | 70.5 ± 17.2 | 0.723 |
| Fibrinogen (g/L) | 3.72 (3.21, 4.68) | 3.62 (3.19, 4.42) | 0.398 |
| Hs-CRP (mg/L) | 4.0 (2.1, 6.0) | 3.4 (2.1, 5.9) | 0.586 |
| Hypertension | 98 (73.1) | 119 (73.5) | 0.950 |
| Diabetes mellitus | 39 (29.1) | 42 (25.9) | 0.541 |
| Dyslipidaemia | 56 (41.8) | 67 (41.4) | 0.940 |
| Smokers | 59 (44.0) | 73 (45.1) | 0.859 |
| Alcohol consumers | 23 (17.2) | 32 (19.8) | 0.569 |
| Hypertension med use | 37 (37.5 ^a^) | 40 (33.6 ^a^) | 0.526 |
| Diabetes med use | 19 (48.7 ^b^) | 21 (50.0 ^b^) | 0.908 |
| NIHSS on day 1 | 4 (2, 7) | 4 (2, 8) | 0.589 |
| NIHSS on day 6 | 3 (1, 7) | / | / |
| mRS scores | 3 (2, 3) | 2 (2, 3) | 0.857 |

**Supplementary Table S1.**

Baseline characteristics of the subgroup of patients have the second blood samples on day 6 after stroke and comparison to the whole LAA stroke patients

Continuous variables are expressed as the mean ± standard deviation (SD) or the median (interquartile range). Categorical values are given as frequencies (percentages).

^a^ represent the percentage in the hypertension population.

^b^ represent the percentage in the diabetes population.

Abbreviations: LAA stroke, large artery atherosclerotic stroke; SBP, systolic blood pressure; DBP, diastolic blood pressure; BMI, body mass index; FBG, fasting blood glucose; TG, triglycerides; TC, total cholesterol; HDL-C, high-density lipoprotein cholesterol; LDL-C, low-density lipoprotein cholesterol; HbA_1C_, glycated haemoglobin; hs-CRP, high-sensitivity C-reactive protein; NIHSS, National Institutes of Health Stroke Scale; mRS, modified Rankin Scale.

| Characteristic | Patients analysed periostin three times  (n = 46) | Patients analysed periostin levels twice  (n = 134) | *p* value |
| --- | --- | --- | --- |
| Age (years) | 70.9 ± 8.1 | 71.4 ± 8.3 | 0.752 |
| Male (%) | 23 (50.0) | 88 (65.7) | 0.151 |
| SBP (mmHg) | 153.3 ± 28.3 | 152.8 ± 24.1 | 0.911 |
| DBP (mmHg) | 81.0 ± 13.4 | 77.9 ± 12.6 | 0.161 |
| BMI (kg/m^2^) | 23.8 ± 4.0 | 23.2 ± 3.4 | 0.354 |
| FBG (mmol/L) | 5.31 (5.00, 7.48) | 5.31 (4.75, 7.29) | 0.395 |
| TG (mmol/L) | 1.23 (1.03, 1.81) | 1.31 (1.00, 1.72) | 0.693 |
| TC (mmol/L) | 4.90 (3.97, 5.52) | 4.34 (3.79, 5.15) | 0.061 |
| HDL-C (mmol/L) | 1.18 ± 0.24 | 1.16 ± 0.22 | 0.506 |
| LDL-C (mmol/L) | 3.14 (2.25, 3.51) | 2.50 (2.11, 3.26) | **0.050** |
| HbA_1C_ (%) | 6.1 (5.8, 7.4) | 5.9 (5.5, 6.4) | 0.070 |
| Homocysteine (μmol/L) | 13.9 (12.1, 17.6) | 12.9 (11.0, 16.4) | 0.179 |
| Serum creatinine (μmol/L) | 69.2 ± 14.6 | 69.8 ± 16.1 | 0.817 |
| Fibrinogen (g/L) | 4.16 (3.44, 5.05) | 3.72 (3.21, 4.68) | 0.086 |
| Hs-CRP (mg/L) | 3.5 (2.3, 6.3) | 4.0 (2.1, 6.0) | 0.662 |
| Hypertension | 36 (78.3) | 98 (73.1) | 0.492 |
| Diabetes mellitus | 18 (39.1) | 39 (29.1) | 0.207 |
| Dyslipidaemia | 24 (52.2) | 56 (41.8) | 0.221 |
| Smokers | 17 (37.0) | 59 (44.0) | 0.402 |
| Alcohol consumers | 8 (17.4) | 23 (17.2) | 0.972 |
| Hypertension med use | 17 (47.2 ^a^) | 37 (37.5 ^a^) | 0.322 |
| Diabetes med use | 8 (44.4 ^b^) | 19 (48.7 ^b^) | 0.764 |
| NIHSS on day 1 | 3 (2, 6) | 4 (2, 7) | 0.182 |
| NIHSS on day 6 | 3 (1, 5) | 3 (1, 7) | 0.133 |
| mRS scores | 3 (2, 3) | 3 (2, 3) | 0.583 |

**Supplementary Table S2.**

Baseline characteristics of the subgroup of patients have the third blood samples on day 6 after stroke and comparison to the patients have the second blood samples on 4th week.

Continuous variables are expressed as the mean ± standard deviation (SD) or the median (interquartile range). Categorical values are given as frequencies (percentages).

^a^ represent the percentage in the hypertension population.

^b^ represent the percentage in the diabetes population.

Abbreviations: LAA stroke, large artery atherosclerotic stroke; SBP, systolic blood pressure; DBP, diastolic blood pressure; BMI, body mass index; FBG, fasting blood glucose; TG, triglycerides; TC, total cholesterol; HDL-C, high-density lipoprotein cholesterol; LDL-C, low-density lipoprotein cholesterol; HbA_1C_, glycated haemoglobin; hs-CRP, high-sensitivity C-reactive protein; NIHSS, National Institutes of Health Stroke Scale; mRS, modified Rankin Scale.
